# *Plasmodium cynomolgi* as Cause of Malaria in Tourist to Southeast Asia, 2018

**DOI:** 10.3201/eid2510.190448

**Published:** 2019-10

**Authors:** Gitte N. Hartmeyer, Christen R. Stensvold, Thilde Fabricius, Ea S. Marmolin, Silje V. Hoegh, Henrik V. Nielsen, Michael Kemp, Lasse S. Vestergaard

**Affiliations:** Odense University Hospital, Odense, Denmark (G.N. Hartmeyer, T. Fabricius, S.V. Hoegh, M. Kemp);; Statens Serum Institut, Copenhagen, Denmark (C.R. Stensvold, H.V. Nielsen, L.S. Vestergaard);; Sygehus Lillebælt, Vejle, Denmark (E.S. Marmolin)

**Keywords:** malaria, *Plasmodium cynomolgi*, human infection, parasites, travel, Southeast Asia, Malaysia, Thailand, Denmark

## Abstract

We report human infection with simian *Plasmodium cynomolgi* in a tourist from Denmark who had visited forested areas in peninsular Malaysia and Thailand in August and September 2018. Because *P. cynomolgi* may go unnoticed by standard malaria diagnostics, this malaria species may be more common in humans than was previously thought.

Despite marked reductions in the global disease burden, malaria remains a serious threat to persons living in or visiting areas to which it is endemic (*1*). Traditionally, 4 species of *Plasmodium* parasites (*P. falciparum, P. vivax, P. ovale,* and *P. malariae*) have been considered to cause natural human malaria; however, several simian *Plasmodium* species have also been found to infect humans (*2*). *P. knowlesi*, a parasite of forest macaques in Southeast Asia, is regularly detected in human malaria cases, including cases involving tourists (*3*). Because of morphological similarity, *P. knowlesi* has been widely misdiagnosed as *P. malariae* or *P. falciparum* by microscopy. In Brazil, *P. simium* was initially identified as *P. vivax* during outbreaks in 2015 and 2016 (*4*), highlighting the need for better methods for accurate identification.

In 2014, another simian *Plasmodium* species, *P. cynomolgi*, was reported to have naturally infected an adult patient (*5*). Until then, *P. cynomolgi* was known as a human parasite only from experimental studies. In addition to fever, clinical symptoms in humans comprise cephalgia, anorexia, myalgia, and nausea; the prepatent period is 7–16 days and the incubation period is ≈15–20 days, with some variation between different strains of *P. cynomolgi* (*2**,**6*).

*P. cynomolgi* is found in long-tailed macaques across Southeast Asia, often concomitant with other simian malaria parasites such as *P. inui*, *P. coatneyi,* or *P. fieldi* (*7*). A recent vector survey in Vietnam demonstrated the presence of *P. cynomolgi* among other human and nonhuman primate *Plasmodium* spp. parasites in *Anopheles dirus*, an important local malaria vector (*8*). Asymptomatic carriage of *P. cynomolgi* was recently reported in village residents in Cambodia (*9*). We report a travel-related case of malaria caused by *P. cynomolgi* in a tourist from Denmark who had traveled to forested areas in peninsular Malaysia and Thailand.

## The Study

A 37-year-old woman from Denmark with no underlying conditions and no previous history of malaria traveled with her husband and children for 6 weeks in various parts of peninsular Malaysia and Thailand in 2018. None of them took malaria chemoprophylaxis; however, they used mosquito repellents and mosquito nets.

The family traveled by air to Singapore in mid-August 2018 and traveled by bus to Kuala Lumpur, Malaysia. From there, they traveled by air to Kota Bharu on the east coast of peninsular Malaysia and sailed to Perhentian Island, staying for 4 days in a beach cottage, with day trips into the nearby forest. In late August, they returned to Kuala Lumpur and traveled by air to Chiang Mai, Thailand, from which they visited remote mountain villages, hiked through the forest, and stayed overnight in local villages. In early September they traveled by air to Bangkok and traveled onward by train to Khao Sok National Park, Surat Thani Province, where they stayed in treehouses in the jungle for 4 nights. In mid-September they traveled by car and ferry to the island Koh Phangan, where they stayed in beach houses for a week. They then sailed to the island Koh Samui for another week of beach holiday before returning to Denmark.

The patient noted numerous macaque monkeys during the jungle visit in Khao Sok, but not in the other areas. She also reported receiving several mosquito bites while in Khao Sok, despite the use of preventive measures.

**Figure 1:**
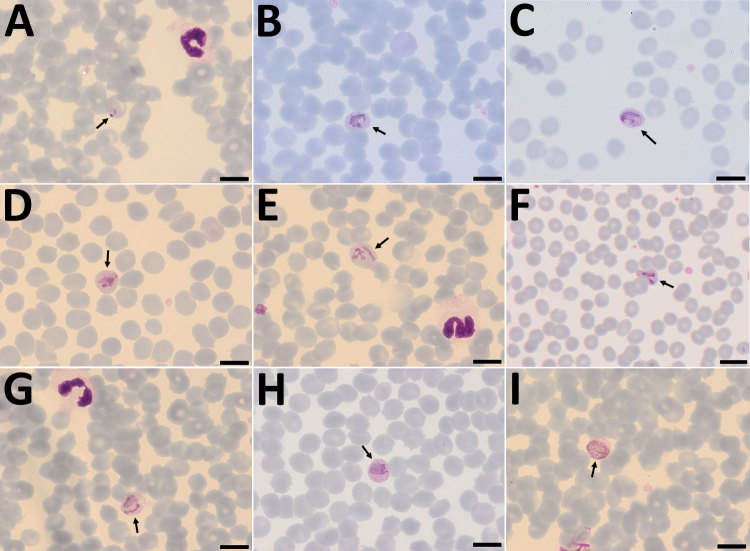
*Plasmodium cynomolgi* parasites (arrows) in Giemsa-stained thin smears of blood from a traveler returning from Southeast Asia to Denmark. Overall, few parasites were visible in the thin film, and no schizonts were visible at all. A) Young trophozoite. The cytoplasm is ring shaped, and the nucleus is spherical. The erythrocyte is not enlarged, and neither Schüffner’s dots nor pigment are visible. B) Growing trophozoite. The young parasite is ring shaped and takes up more than half of the diameter of the host erythrocyte. The cytoplasm has become slightly amoeboid. Schüffner’s dots are more prominent than in *P. vivax* at this stage. Pigment is visible as small yellowish granules in the cytoplasm. Erythrocyte enlargement is not evident. C) Growing trophozoite. The cytoplasm appears amoeboid but relatively compact. Schüffner’s dots are prominent, but no pigment is seen in the cytoplasm. The erythrocyte is slightly enlarged. D) Growing trophozoite. The cytoplasm appears amoeboid, and the nucleus has increased in size. Schüffner’s dots and yellowish pigment are prominent. Enlargement of the erythrocyte is evident. E) Growing trophozoite. The host cell is further enlarged. The cytoplasm is amoeboid as in *P. vivax* at this stage. Schüffner’s dots are clearly visible, and yellowish pigment is dispersed within the cytoplasm. F) Growing trophozoite. An infected erythrocyte with major alteration in the shape, similar to that sometimes seen in *P. vivax–*infected erythrocytes. The cytoplasm is amoeboid, with hardly any pigment. Schüffner’s dots are prominent, and the host erythrocyte is enlarged. G) Growing trophozoite. The cytoplasm is amoeboid and appears relatively compact. Schüffner’s dots are dominant. Pigment is visible in small granules but appears more yellowish-brown and is scattered around in the cytoplasm. H) Near-mature trophozoite. The parasite is becoming more compact with an enlarged nucleus. No ring or amoeboid form is visible. Schüffner’s dots are very dense, and abundant yellowish-brown pigment is clearly visible in the cytoplasm. I) Mature microgametocyte. It is round and resembles that of *P. vivax* at the same stage. The nucleus is diffuse and takes up most of the parasite. The stippling of the host cell is forced toward the periphery, as seen for *P. vivax.* Microgametocytes stain reddish-purple (pink hue) in contrast to macrogametocytes, which stain light blue. The yellowish-brown pigment is scattered around in the parasite. Scale bars indicate 100 μm.

The day before returning to Denmark, the patient experienced muscle pain, general malaise, fever, headache, and abdominal pain. Symptoms gradually declined after 6–8 hours but returned after 12 hours. This cyclic pattern repeated 5–6 times. On the day after her return, she was admitted to the local hospital for fever (39.0°C) and suspected malaria; circulatory and respiratory systems were stable. Chest radiograph results were normal. Blood examination showed slightly elevated C-reactive protein (39 mg/L), leukocyte count within reference range (6.3 × 10^9^ cells/L), slightly elevated S-alanine aminotransferase (S-ALAT, 55 IU/L), and thrombocytopenia (104 × 10^9^ platelets/L); platelet levels on the next day further dropped to 96 × 10^9^ /L. Results of initial malaria testing using a malaria rapid diagnostic test (RDT) (First Response Malaria Ag Combo Test, Premier Medical Corporation Ltd., http://premiermedcorp.com) and malaria microscopy were negative. Because her symptoms persisted, we tested an EDTA-blood sample by loop-mediated isothermal amplification (Illumigene Malaria; Meridian Bioscience, https://www.meridianbioscience.com); results were positive for *Plasmodium* spp. Repeated microscopic examination of Giemsa-stained thin blood smear (10%, pH 7.2 for 15 min) revealed low-grade parasitemia. We observed different parasite stages that resembled *P. vivax* but with slightly different morphologic features (Figure 1) (*2*).

**Figure 2:**
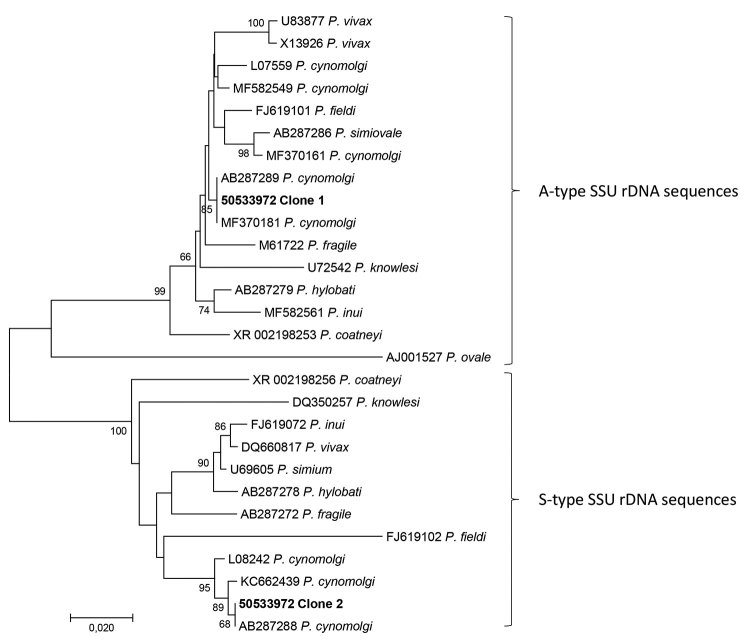
Phylogenetic analysis of the 2 consensus sequences (50533972 clone 1 and 50533972 clone 2) generated by the microbiome assay of blood from a traveler returning from Southeast Asia to Denmark. We used CD-HIT Suite (http://weizhong-lab.ucsd.edu/cdhit_suite/cgi-bin/index.cgi?cmd=cd-hit-est) to cluster sequences reflecting *Plasmodium*-specific DNA amplified and sequenced by our microbiome assay; we generated consensus sequences using an in-house sequence clustering software. We queried the 2 resulting consensus sequences in GenBank, then downloaded examples of DNA sequences with varying genetic similarity and included them in a multiple sequence alignment with the 2 consensus sequences. Phylogenetic analysis revealed that the microbiome assay had amplified asexual stage-specific (A-type) SSU rRNA genes of *Plasmodium cynomolgi*, with 50533972 clone 1 reflecting them, and sporozoite stage-specific (S-type), with 50533972 clone 2 reflecting them. We conducted phylogenetic analysis involving 28 DNA sequences in MEGA7 (http:/www.megasoftware.net) and included a total of 464 positions in the final dataset. We inferred evolutionary history using the neighbor-joining method. Numbers at the branches show the percentage of replicate trees in which the associated taxa clustered together in the bootstrap test (1,000 replicates). The tree is drawn to scale, with branch lengths in the same units as those of the evolutionary distances used to infer the phylogenetic tree. We computed evolutionary distances using the Kimura 2-parameter method. Scale bar indicates nucleotide substitutions per site.

We referred the patient to a tertiary hospital for treatment and follow-up. Repeated LAMP was positive for *Plasmodium* DNA, whereas the rapid test was again negative. In-house real-time PCRs were positive for *Plasmodium* (*10*) but negative for *P. falciparum* (*11*), *P. vivax* (*11**,**12*), *P. ovale* (*13*), *P. malariae* (*13*), and *P. knowlesi* (*14*). A blood sample was analyzed at the National Reference Parasitology Laboratory by PCR using genus-specific primers (Plasmo F 5′-TTGYCTAAAATACTTCCATTAATCAAGAACG-3′ and Plasmo R 5′-TTTGATTTCTCATAAGGYACTGAAGG-3′) and a next-generation sequencing–based (NGS) microbiota assay, described previously (*15*). Sanger sequencing of the genus-specific PCR product revealed *P. cynomologi*. The assay identified 2 stage-specific types of nuclear small subunit (SSU) rRNA genes (*16*), both belonging to *P. cynomolgi*. Clone 1 exhibited 99.51% similarity to GenBank accession no. AB287289 (asexual [A]–type SSU rDNA), and clone 2 had 100% similarity to GenBank accession no. AB287288 (sporozoite [S]–type SSU rDNA), both of which were isolates identified in a long-tailed macaque in Southeast Asia (Figure 2).

The patient received atovaquone/proguanil (1,000/400 mg/d for 4 d), followed by primaquine (26.4 mg/d for 14 d). Symptoms resolved on the second day of treatment, and the patient was discharged for outpatient follow-up. Within a week, platelet count normalized, and S-ALAT further increased to 135 U/L. Results of malaria microscopies repeated on days 9 and 37 of treatment were negative. The patient fully recovered.

## Conclusions

A short-term traveler contracted *P. cynomolgi* malaria during a trip to Southeast Asia. Exactly where the patient became infected is not known. The presence of nocturnal mosquitoes and macaques makes Khao Sok National Park in Thailand a likely site of infection. The time interval of 17 days between her arrival in Khao Sok and the onset of symptoms matches reported incubation periods for human *P. cynomolgi* infections (*6*).

Surat Thani Province in Thailand is located ≈800 km north of Hulu Terengganu in peninsular Malaysia, where a natural human *P. cynomolgi* infection was only recently reported in a local resident (*5*). Long-tailed macaques are present in both of these areas, but not around Chiang Mai (*3*).

We obtained species identification by DNA sequencing only after negative species-specific real-time PCR. Given the challenge of diagnosing *P. cynomolgi* and the widespread occurrence of its natural host across Southeast Asia, it is likely that this simian *Plasmodium* sp. is underdiagnosed in both residents and visiting travelers.

Urban development into forested areas leads to closer coexistence of human and monkey. The number of cases in which malaria is transmitted from monkeys to humans may therefore increase. Advanced detection and identification techniques may improve knowledge of the epidemiology of simian malaria in humans.
